# Judge Scores Dental School. Refuses Mandamus for License to a German-American College Graduate

**Published:** 1902-11-15

**Authors:** 


### JUDGE SCORES DENTAL SCHOOL. REFUSES MANDAMUS FOR LICENSE TO A GERMAN-AMERICAN COLLEGE GRADUATE.

The petition of Etienne Stump for a writ of mandamus to compel the State board of dental examiners to issue him a license to practice, was dismissed yesterday by Judge Chetlain. The judge scored the German-American Dental College in giving the reasons for his decision. He was particularly severe with Dr. Huxmann, the dean of the school.

April 30, 1902, the State board refused to license Stump on the ground that the German-American Dental College, of which he is a graduate, was not an institution of sufficient standing and repute to warrant it. The result was the prayer for mandamus charging the board with improper motives.

In giving his decision Judge Chetlain said: "It appears from the evidence that Dr. Huxmann is, and has been the head and front of the institution. It appears there were no meetings of the faculty and no regular books were kept, and when counsel for the respondent asked for the book containing the names of the students who had matriculated, it was not produced. The attitude of Dr. Huxmann was inconsistent. While claiming the benefit of the law, he utterly ignored the regulations of the board, sending out prospectuses since the adoption of the rules of October 18, 1901, showing a course of study of only three successive semesters of six months each.

"Moreover, if the testimony of some witnesses is to be believed, Dr. Huxmann also violated the rules of the board by promising speedy graduation to some of his students contrary to the printed requirements of his own college as well as of the board, and during an examination held by the State board he furnished the answers to questions to his students in advance.

"The evidence also shows that part of the time for the last ten years Dr. Huxmann was himself a member of the board, and a part of that time was translator for the board of the examination papers from his own college, and that he continuously sought and availed himself of advantages and privileges not accorded to other colleges, in direct violation of the rules of the board.

"Whether these concessions were the result of sinister influence or the unsolicited favors of the generous board, we are left to conjecture. They were at least illegal and unjust."—*Chicago Tribune*, Aug. 10, 1902.

